# Sex Hormone Receptor Repertoire in Breast Cancer

**DOI:** 10.1155/2013/284036

**Published:** 2013-11-13

**Authors:** Gerald M. Higa, Ryan G. Fell

**Affiliations:** ^1^Schools of Pharmacy and Medicine, West Virginia University, Morgantown, WV 26506, USA; ^2^Robert C. Byrd Health Sciences Center, West Virginia University, One Medical Center Drive, P.O. Box 9520, Morgantown, WV 26506, USA; ^3^School of Pharmacy, West Virginia University, Morgantown, WV 26506, USA

## Abstract

Classification of breast cancer as endocrine sensitive, hormone dependent, or estrogen receptor (ER) positive refers singularly to ER**α**. One of the oldest recognized tumor targets, disruption of ER**α**-mediated signaling, is believed to be the mechanistic mode of action for all hormonal interventions used in treating this disease. Whereas ER**α** is widely accepted as the single most important predictive factor (for response to endocrine therapy), the presence of the receptor in tumor cells is also of prognostic value. Even though the clinical relevance of the two other sex hormone receptors, namely, ER**β** and the androgen receptor remains unclear, two discordant phenomena observed in hormone-dependent breast cancers could be causally related to ER**β**-mediated effects and androgenic actions. Nonetheless, our understanding of regulatory molecules and resistance mechanisms remains incomplete, further compromising our ability to develop novel therapeutic strategies that could improve disease outcomes. This review focuses on the receptor-mediated actions of the sex hormones in breast cancer.

## 1. Introduction

Epidemiological, biological, and clinical data strongly implicate the role of sex hormones, primarily estrogens, in breast cancer; yet, their mere presence does not contribute to the malignant process. Inherent in this technically accurate paradox is that while the former supports the well-established link between estrogens, and possibly androgens, in this endocrine-related cancer, the latter infers that generation of the malignant phenotype requires other cellular components. Perhaps the most important “other” element is the hormone receptor. While the ligand/receptor construct is conceptually very simple, the molecular mechanisms by which sex hormones regulate a number of dynamic yet delicate processes in their target tissues are exceedingly more complex.

The impetus for undertaking this endeavor was to merge our increased, though by no means complete, understanding of the estrogen and androgen receptors in breast cancer. As such, numerous published papers, some old but of enduring scientific value, were evaluated to support the conclusion that the nuclear steroid receptors are the essential link between hormone and disease. And to further enhance reader appreciation of this complex structure, the biology of the receptor is briefly reviewed in order to provide additional insight into the proposed mechanisms which promote tumor growth as well as facilitate tumor resistance. Here, the reader can learn a little about the treatment of hormone-dependent breast cancer, more about how cell biology might improve our understanding of this disease, and perhaps better appreciate the role receptors have in a disease that may be more appropriately referred to as sex hormone receptor-dependent breast cancer.

## 2. Evolving Principles

Although an association between estrogens and breast cancer was recognized over a century ago [1], a valid explanation for how estrogens exert their tissue-specific actions was not forthcoming for another 60 years. Rather than their speculated role in enzyme-mediated metabolic processes, a protein receptor was identified as the critical component to mediate the broad repertoire of hormonal actions including estrogen-stimulated growth of breast cancer cells [2]. What later became the target for new strategies to treat breast cancer, the estrogen receptor (ER) was also on the verge of being associated with the disease in two defining ways. First, as a predictive factor, in that the ER emerged as the single most useful determinant to guide application of (and likely, response to) endocrine therapies [3]; and second, as a prognostic factor, in that independent of treatment, tumor cell expression of this hormone receptor was associated with improved disease outcomes [4]. More recently, the ER has been linked to two molecular subtypes of breast cancer identified as luminal A and luminal B [5]. Further testing of ER-positive (ER+) tumors by analyzing expression of a panel of genes resulted in groundbreaking information for managing women with early-stage hormone-dependent breast cancer [6]. Translated to a “recurrence score,” the multigene test had, like ER, both prognostic value (quantified the risk of disease recurrence) and predictive value (estimated the benefit of adding chemotherapy).

These noteworthy attributes may have, in large part, pre-empted the potential importance of the second ER, which was discovered less than two decades ago [7]. Because of this new finding, what was traditionally referred to as ER, subsequently became known as ER*α*. Even though accumulating data suggest the more recently identified estrogen receptor (i.e., ER*β*) may have biological and clinical relevance, the majority of the published reports support the preeminence of ER*α*, even in tumor cells that coexpress both ER subtypes. While this conclusion is consistent with reports that breast cancer survival was not influenced by ER*β* expression in patients with ER*α*+ or ER*α*-negative (ER−) tumors [8, 9], other investigators found a direct correlation between ER*β*-positivity and overall survival [10]. A confounding factor that could provide a partial explanation for this discrepancy is the variable expression of one or more ER*β* variants in breast cancer (Table 1). Because of the potential clinical relevance of ER*β*, a comprehensive discussion of this issue is provided in the ER*β* section which follows later.

Interestingly, expression of the androgen receptor (AR) in primary breast tumors and metastatic lesions predated the discovery of ER*β* [11]. However, like ER*β*, the clinical significance of its presence in tumor cells remains uncertain. Nevertheless, a number of studies suggest that the AR may have prognostic value, independent of ER*α* expression. For example, even though AR is frequently present in ER*α*+ breast cancers, AR has also been found in a significant percentage of ER*α*− tumors [12, 13]. These observations may be clinically relevant for the following reason. Because tumor cell expression of ER*α* correlates with tumor size, histologic grade, and axillary node metastasis, it may be difficult to argue that the relatively good prognosis of ER*α*+ breast cancers should be attributed, in part, to coexpressed AR. However, the finding that expression of AR in ER*α*− tumors was associated with increased age, postmenopausal status, lower tumor grade, smaller tumor size, and significantly improved disease-free survival suggests otherwise [13]. Moreover, the absence of AR appeared to be associated with higher levels of Ki-67, a cell proliferation marker. Collectively, these data highlight the possibility that sole expression of AR in tumor cells may not only be a prognostic indicator but also a therapeutic target [14]. 

What may be most important are the actions mediated through each receptor (or the confluence of receptor-mediated actions) on tumor cells and, ultimately, on disease outcomes. Hence, a better understanding of the biology of these receptors may provide part of the answer.

## 3. Receptor Biology

**Figure 1:**
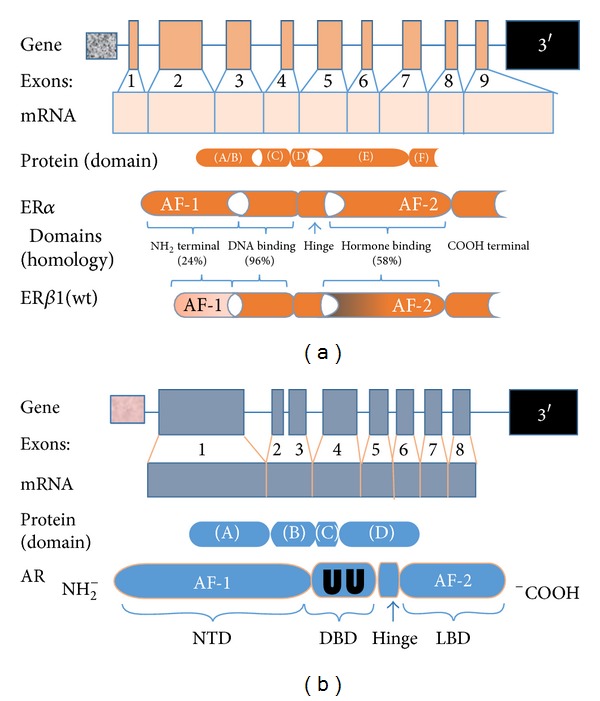
(a) Characterization of the estrogen receptor gene and protein. ER*α* and ER*β* are encoded by two distinct genes; the ER*α* gene is localized to chromosome 6q24–27; the ER*β* gene is located on chromosome 14q21-22. The gene transcripts are composed of 9 exons. The two encoded proteins differ in number of amino acids with ER*α* being slightly longer, 595 versus 530. Both proteins have five distinct domains, three of which have relative degrees of homology. The domain with the greatest disparity resides in the A/B domain, which may account for many of the antagonistic actions observed between the two ERs. (b) Characterization of the androgen receptor gene and protein. The human androgen receptor gene is situated on the long arm of the *X-chromosome, q11-12. *The organization of the protein coding region is similar to that of the estrogen receptor but is divided over 8 exons. The sequence encoding the N-terminal domain (NTD) is found in exon 1. Similar to the ER, the functionality of the DNA-binding domain (DBD) is provided by a motif comprised of two zinc fingers (UU) encoded by exons 2 and 3. The first zinc finger mediates DNA recognition while the second mediates DNA-dependent dimerization. Message for the ligand-binding domain (LBD) is distributed over the remaining five exons.

The sex hormone receptors, which belong to the steroid/thyroid superfamily of nuclear receptors, mediate the genomic action of estrogens by acting as ligand-dependent transcription factors [15]. All members of the hormone-inducible nuclear receptors share three distinct but not autonomously functioning domains (Figure 1). First, although the NH_2_-terminal domain (NTD) contains activating factor (AF)-1, which serves as the foci for transcription factor interaction and target-gene activation, the activities of the AF-1 domain differ substantially between the two ERs [16]. The nature of this disparity is discussed in the ER*α*/ER*β* heterodimer section. Next, the DNA-binding domain (DBD) contains two highly conserved zinc-finger regions that are essential for high-affinity binding to estrogen response elements (EREs) in target genes and comodulating receptor dimerization with the ligand-binding domain (LBD). And third, the C-terminus domain mediates ligand-binding, receptor dimerization, and nuclear translocation. Localized to the latter domain is AF-2.

 Although having similar functional domains, a critical component of signaling mediated through AR depends on receptor dimerization, of which three forms have been described. There is convincing evidence that dimerization mediated through the DBD is perhaps the most important [17, 18]. Of note, the DBD of both AR and ER is enriched with two cysteine zinc finger motifs and a C-terminal extension (CTE) [19]. However, in contrast to the ERs, the first 12 amino acids of the CTE are essential for AR-specific DNA binding [20, 21]. A second form of AR dimerization occurs through an interaction between two discrete sites in the N-terminal and C-terminal (N/C) [22]. Induced only by AR agonists such as 5*α*-dihydrotestosterone and testosterone, the N/C interaction stabilizes the receptor by slowing the rate of steroid dissociation and receptor degradation [23, 24]. Furthermore, the N/C interaction appears to be a critical regulator of chromatin binding and transcription [25]. Lastly, compared to LBD-mediated dimerization (the third form) of ER, much less is known about the specific sites involved with AR [26]. Collectively, our current knowledge strongly suggests that dimerization processed through the DBD is an absolute requirement for AR-mediated signaling.

**Figure 2:**
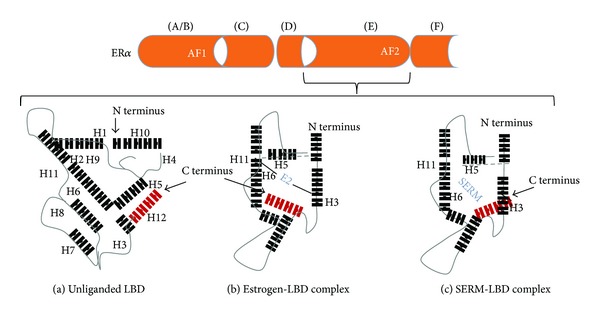
Monomeric modeling of ER*α* LBD. The LBD (a) is a three-dimensional configuration composed of three folded strata. The central core is comprised of helices 5, 6, 9, and 10, which is positioned between two layers, one composed of helices 1–4; the other helices 7, 8, and 11. The size of the binding cavity is approximately two-fold greater than the molecular volume of estradiol. E2 binds diagonally (b) between helices 11, 6, and 3 and induces a conformational change in the LBD. Devoid of contact with the ligand, helix 12 (in red) is repositioned, thus providing a protective seal over the binding cavity. Reorientation of helix 12 in this manner intrinsically generates AF2, recruits coactivators, and promotes transcription. However, when the receptor binds selective estrogen receptor modulator or SERM (c), the length of the SERMs side chain exceeds the confines of the binding cavity. As a result, helix 12 is misaligned over the binding pocket. Diffraction studies indicate helix 12 is rotated 130° toward the N terminus of the LBD.

Even though our understanding of the receptor multiplex continues to evolve, construction of exquisite three-dimensional models has made it possible to better appreciate and even visualize the submicroscopic precision of the hormone/receptor interaction. The primal event of ligand binding at the C-terminal end induces conformational changes in the receptor and activates intrinsic AF-2. Crystallographic studies of the LBD revealed that the AF-2 interaction platform is composed of five helices, the most important of which appears to be positioning of helix 12. When estradiol binds ER*α*, helix 12 is oriented over the ligand-binding pocket creating an “agonist” interface for recruitment of coactivators (Figure 2) [27]. On the other hand, the tamoxifen/ER*α* complex results in displacement of helix 12 from its agonist position and, instead, occupies the lipophilic pocket formed by helices 3, 5/6, and 11. In this context helix 12 disrupts the coactivator interactive surface. Hence, ligand-induced reorientation of helix 12 is not only an important feature which discriminates ER agonists from antagonists but also essential for receptor-mediated transcriptional activity. Similarly, androgen-bound receptor promotes conformational rearrangements in the LBD resulting in formation of AF-2 [28]. Interesting however, unlike ER AF-2 which is the principal mediator of transcriptional activity through coregulator binding in the LBD, the AR AF-2 domain preferentially interacts with amino acid motifs found in the N-terminal domain. As such, recruited and bound coregulators make the NTD of AR the primary site of transcriptional activity [29, 30].

**Figure 3:**
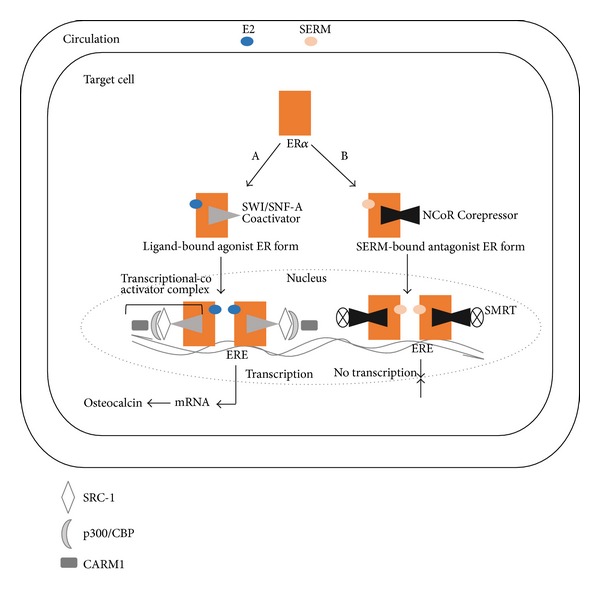
Schematic representation of the genomic effect of estradiol. Nuclear translocation of ligand-bound receptor. (A) Activation of targeted genes in osteoblasts necessitates recruitment of two chromatin remodeling complexes known as SWI/SNF-A (switching defective/sucrose nonfermenting), as well as coactivators of the p160 family including SRC-1, SRC-2, and SRC-3 (steroid receptor coactivators 1, 2, and 3). All of the SRCs possess three nuclear receptor (NR) boxes located in the receptor interacting domain, which enables direct interaction with ER*α*; and two activation domains, AD1 and AD2, which serve as binding sites for p300/CBP (cointegrator-associated protein), and CARM1 (coactivator-associated arginine methyltransferase 1). The importance of these coregulators, especially p300/CBP, relates to the latter's interaction with the AF-2 domain of the ERs, which prompts recruitment of histone acetylases to the receptor. Together with the SRC complexes, the epigenetic enzyme disrupts DNA stability, thus allowing transcription of the target genes. (B) Repression of transcription occurs in a manner opposite to that of activation. Upon SERM binding, the receptor undergoes conformational changes that enhance interactions with corepressors. Three of the most well-known repressors of ER*α*-mediated transcriptional activity are NCoR (nuclear corepressor), silencing mediator of retinoid and thyroid receptor (SMRT), and repressor of ER*α* activity (REA). This transcriptional comodulatory apparatus results in inhibition of transcription.

In contrast, the molecular basis of how the hormone-bound receptor interacts with the nuclear transcriptional elements makes the ligand binding process look relatively simple as recruitment of additional coregulators and involvement of epigenetic proteins has been shown to be essential in order to fine tune hormonal activity [31]. In addition, transcription can be activated or repressed depending on the recruitment, or balance, of the comodulatory components. Examples of both types of transcriptional activity are illustrated using estrogen actions on the skeleton as osteocytes contain ER*α* (Figure 3). Whereas many of the genes that affect bone metabolism have been identified, the manner in which transcription takes place depends on surreptitiously precise interactions with a number of coactivator complexes.

Although negative feedback is a unique biological characteristic of the endocrine system, molecular evidence suggests that part of the mechanism for self-attenuation or inhibition of receptor-mediated signaling depends on the presence of corepressors (Figure 3). As indicated previously, estrogen-induced rotation of helix 12 within the AF-2 domain facilitates coactivator binding. However, in the presence of the anti-estrogens tamoxifen and raloxifine, an alternative conformation in the LBD of ER in which helix 12 blocks the coactivator recognition site results which favors corepressor binding [27]. 

In essence, the ultimate effect of hormones on target tissue depends on the balance of coactivators and corepressors recruited to the receptor. This concept is relevant in breast cancer because receptor-mediated effects appear to result from both upregulation of specific genes that contribute to tumor growth and survival as well as downregulation of genes that possess repressive effects on proliferation and apoptosis. Indeed, ER*α*-targeted gene expression can be downregulated in the presence of receptor corepressors which consequently blunt the effect of estrogens on tumor cell proliferation [32]. While the impact of corepressors may be restricted to receptor-expressing (i.e., ER*α*+) and hormone-dependent tumor cells, the development of hormone-independent breast cancers could also be partly linked to diminished corepressor activity. This conclusion is supported by the following: (1) it has been shown that corepressors bound to un-liganded ER*α* are dislodged by estradiol [33]; and (2) these same corepressor complexes, which are recruited by antiestrogens, could conceivably contribute to some of tamoxifen's antitumor effect [34].

Summation of these data indicates that activation or repression of ER*α*-mediated DNA transcription depends on multiple factors, all of which appear to be contingent on the type of ligand/receptor complex formed, as well as the balance between types of comodulatory proteins recruited. In essence, an intrinsic cellular program mediated through the hormone receptors follows a blueprint for the repetitive remodeling of chromatin, regulates the process of gene transcription, and determines specific endocrine effects on target tissue. The importance of the above information is discussed further in sections related to the individual receptors.

## 4. Sex Hormone Receptors

### 4.1. ER*α*

Not by default related to its early discovery, but rather by de facto evidence, both laboratory and clinical data support the conclusion that estrogenic actions in normal breast, as well as in breast cancer, are mediated primarily through ER*α*. Interestingly, the effect on mammary gland development may be direct or indirect. Compelling evidence of a direct effect comes from the finding that compared to normal mice ER*α*-knockout (*α*ERKO) mice retain only rudimentary mammary ductal structures despite elevated levels of circulating estradiol [35]. The importance of the receptor being established led to the intriguing question whether oncogene-induced mammary tumors would still develop in *α*ERKO mice. Even though tumor-inducing amplified Human EGFR-Related (HER) *2* could still promote mammary carcinogenesis in this estrogen-insensitive milieu, devoid of ER*α*, onset of tumor appearance was significantly delayed [36]. These data imply that while the presence of ER*α* was not absolutely essential for tumor development, absence of signaling through the hormone receptor transiently repressed tumor promotion or progression. Estrogens may also have an indirect effect on breast tissue by upregulating a number of growth factor genes including *epidermal growth factor and insulin-like growth factor-1*, both of which may influence, or have a contributory role in, the disease [37, 38]. 

Adding to, or possibly superseding, the importance of tissue ER*α* expression are the target genes regulated by the receptor. Activated ER*α* induces transcription of a vast array of genes, which provide valuable insight into the broad range of tumor cell responses that can be mediated through the receptor. Depending on the type, or balance, of comodulators recruited to the LBD, the mitogenic effects of estrogens are attributable not only to regulating tumor cell proliferation but also suppressing tumor cell apoptosis. Laboratory evidence indicates that ER*α* affects transcription of proproliferative genes such as *proliferating cell nuclear antigen* (*PCNA*) and antiapoptotic genes including *survivin and Bcl* (B-cell leukemia)-2 [39]. One of the more intriguing estrogen-responsive genes is *WNT11 *(a portmanteau of Wg (wingless) in *Drosophila and INT 1 *(the mammalian homologue of Wg)) [40]. Although there is a strong association between *Wnt11* gene expression and hormone-independent growth of prostate cancer cells, its role in breast cancer may not be through Wnt11-mediated tumor cell proliferation but rather inhibition of apoptosis.

It is important to emphasize that the list of ER*α* target genes mentioned above is by no means exhaustive as other types of genes and their individual role in signal transduction, transcription coactivation, and metabolic enzyme processes are also likely to be relevant but were not included. In addition, the genomic actions of estrogen can be further complicated when ER*β* is coexpressed in the same cell.

### 4.2. ER*β*

Regardless whether the terms endocrine responsive, hormone dependent, or ER positive are used to classify a specific subtype of breast cancer, all three descriptors refer singularly to ER*α*. Notwithstanding ER*α*'s distinctive status, data continue to accumulate regarding the potential relevance of the second estrogen receptor. Perhaps the most significant biological difference between the two receptors is the growth suppressive effects ER*β* imparts on breast cancer cells [41–44]. An ingenious *in vitro *study conducted using ER*β*-transfected MCF-7 (ER*α*-positive) cells demonstrated the impact on estrogen-mediated cellular responses when both receptors are expressed. In order to establish expression of the approximate percentage of ER*β* to ER*α* (i.e., 30%) observed in most breast cancers, cells were infected with different numbers of ER*β* adenovirus [41]. By doing this, the investigators were also able to assess the effect different levels of ER*β* expression had on endogenous ER*α* protein levels. Notably, ER*α* protein levels decreased significantly in cells where ER*β* expression exceeded that ER*α*, while expression of ER*α* was unchanged in cells that had the appropriate ratio of receptors.

Interestingly ER*β*-induced suppression of tumor cell proliferation has been observed to occur with or without estrogen [42]. Compared to control tumor cells, induced expression of ER*β* in MCF-7 cells cultured with estradiol resulted in a dramatic reduction in cell growth. Of note also, a reduction in the basal growth rate was observed in ER*β* expressing MCF-7 cells (compared to control tumor cells) in media devoid of ligand. Transcript analysis of the cells provided some plausible mechanisms for the suppressive effects of ER*β*. Several proteins involved in cell cycle progression/cell proliferation including CDC25A (cell division cycle 25 homolog A), the E2F1 transcription factor, and the antiapoptotic protein, survivin, were all downregulated by ER*β*. In addition, p21^WAF1^, a negative regulator of the cell cycle, was upregulated by ER*β*. Collectively, these findings indicated that the observed effect on MCF-7 cells occurred by modulating both positive and negative regulators of cell growth.

In addition to affecting tumor cell proliferation and survival, lower expression of ER*β* in breast cancer (compared to normal breast) suggests the receptor may also have a role in the tumorigenic process. Engineered receptor-null MDA-MB-231 breast cancer cells were transfected with either ER*α* or ER*β* [43]. In the presence of estrogen, both proteins underwent nuclear translocation and were transcriptionally active. However, contrary to their (similar) effect on several estrogen-targeted genes, only ER*α* could induce the proto-oncogene *c-myc*. Hence, in contrast to the proliferative effect of ER*α*, ER*β*-mediated transcriptional activation appears to be protective against hormone-dependent breast cancers.

**Figure 4:**
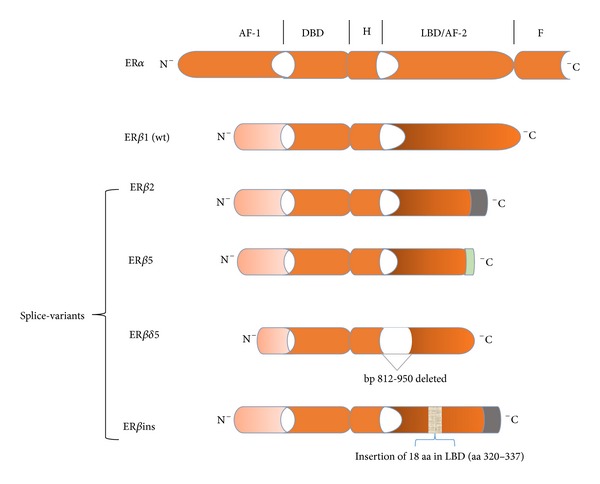
ER*β* splice variants. The AF1 and AF2 domains exhibit the least homology between ER*β*1 and ER*α*. ER*β*2 is nearly identical to ER*β*1 except that the last 61 amino acids (aa) are replaced by 26 novel aa (indicated by the green portion of the receptor). As such, the *β*2 splice variant is comprised of 495 aa. Similarly, the last 65 aa of ER*β*5 has been replaced by 7 other aa resulting in a 472 aa protein. Of note, truncation of the LBD/AF1 domains of *β*2 and *β*5 results in loss of ligand-binding activity, while conservation of their DBD maintains the likelihood of ERE binding. Estrogen binding to the delta5 splice variant is compromised by deletion of nucleotides 812–950 (139 base pairs (bp)) in the LBD. The result of the 18 aa insertion into what is essentially ER*β*2 markedly diminishes ligand binding.

Although coexpression of both ER*α* and ER*β* has been commonly observed, two studies found that approximately half of all ER*α*− breast tumors expressed ER*β* [8, 45]. Of interest also, the absence of ER*α*, which characterizes two other molecular breast cancer phenotypes (i.e., HER2 and basal-like), somewhat surprisingly expressed ER*β* in a substantial number of tumor specimens [8]. Even though the full implications are not known, an inverse relationship between prognostic features such as tumor grade, tumor size, and node involvement and ER*β* expression has been reported [8]. In addition, the presence of ER*β* has been found to correlate with improvements in a number of disease endpoints including relapse-free survival and overall survival [9, 10]; however, conflicting data have also been reported [46, 47]. Because of the potential value of measuring ER*β*, it is important to reconcile these disparate clinical observations. Even though numerous publications suggest that the actions of ER*β* are markedly different from ER*α*, with the former functioning as a tumor suppressor, one major confounding factor that could partially explain the discordant findings may be related to ER*β* variants of which (other than wild type) at least four have been identified (Figure 4). Because many studies analyzed ER*β* at the transcript level using primer sequences common to all variants, identification of what may be functionally distinct isoforms of the receptor was not ascertained [48, 49]. For example, while estrogen binds to ER*β*1 (wild type) [50] and induces transcription of anti-proliferative and pro-apoptotic genes none of the other four variants exhibit high ligand binding affinity. Hence, the apparent protective effect of ER*β*1 against ER*α*-dependent breast tumors may not extend to hormone-dependent tumors that coexpress express ER*β*2 or ER*β*5 [42, 51]. Transcriptional activity also depends on the each variant's ability to bind EREs; much uncertainty exists in this regard with ER*β*5 and ER*δ*5 [52, 53]. Other peculiarities that could add to the inconsistent findings include the relatively small numbers of patients studied and immunohistochemical detection of only ER*β*1 in tumor samples; the primary reason for the latter is often attributed to lack of variant-specific antibodies [54]. Finally, what may be extremely important is the dominant negative activity of ER*β*1, ER*β*2, and ER*β*ins against ER*α* which is discussed in the following section.

### 4.3. ER*α*/ER*β*1 Heterodimer

Even though the ERs are encoded by two different genes [55], the receptors share a number of similarities. For example, the homology of the DNA (C) binding domain is nearly identical, their transactivation properties are similar, and both receptors bind to consensus ERE. Nonetheless, and despite some overlapping gene regulatory activities substantial structural and functional differences between the two transcription factors have been reported [16]. Of the five major domains, the greatest between-receptor disparities occur in the N-terminus (A/B domain) and ligand-binding (E) domains. The relevance of these findings relates to transcriptional AF-1 and AF-2, which are localized to the A/B and E domains, respectively. Furthermore, AF-1 and AF-2 determine both cell- and promoter-specific activities as well as interactions with comodulatory proteins [16]. In addition each receptor's dependence on AF-2 is remarkably different. Whereas AF-1 of ER*α* can function independently of ligand and AF-2, that of ER*β*1 appears totally dependent on ligand-activated AF-2. Moreover, when AF-1 and AF-2 are both activated, the functional activity of homodimeric ER*α* predominates over the ER*β*1 homodimer [16]. 

However, another aspect of the dimer concept completely debunks the notion of ER*β*'s subservient role in relation to ER*α*-mediated transcriptional activity. Whereas genomic actions of estrogens resulting in tumor cell proliferation and survival have been shown to occur via homodimeric ER*α*, there is the distinct possibility that some of the ER*β* variants could antagonize these effects through heterodimerization [56–59]. Indeed, ER*α*/ER*β*1 heterodimers have been localized to specific DNA binding regions of a number of genes implicated in cell proliferation [60]. Notable also, not only are some ER*α*-inducible genes downregulated by the *α*/*β*1 heterodimer, but also increased transcription of genes not affected by either homodimer [61, 62]. However, one of the more confounding issues related to ER*β* involves the formation of ER*α*/ER*β*-splice variant heterodimers. Because tamoxifen has pure antagonist effects on ER*β*1, the finding that a greater proportion of ER*β*2-negative tumors responded to the antiestrogen implies that expression of ER*β*2 is associated with poor response and hence clinical outcomes. This notion is certainly consistent with the concept that antitumor effects achieved through ER*α* blockade could be partially counteracted by inhibiting the growth inhibitory effects mediated by ER*β*2 [49]. On the other hand, some studies have found that expression of ER*β*2 was associated with significant improvement in relapse-free survival and overall survival [63, 64]. Though the findings are inconsistent, the clinical endpoints may be independent of tamoxifen's effect on ER*β*2 (as well as formation of the *α*/*β*1 heterodimer) because of altered ligand binding due to changes in the truncated C-terminus of the protein and loss of the AF-2 domain [65]. 

Of interest also is the expression of the progesterone receptor (PR), which is known to be regulated by ER*α* [66]. Because the presence of ER*β*1 has been shown to repress transactivation of the *PR *gene [67], the heterodimer, in a manner similar to its antiproliferative effects, could also suppress this ER*α*-target gene. Moreover, this finding could explain the clinical phenomenon of ER+/PR− breast cancers. These findings suggest not only that genomic action (and hence, biological effect) of estrogens differs depending on receptor dimeric state but also the possibility of a reciprocal influence each receptor has on the other (Table 1).

### 4.4. AR

The finding that expression of AR is ubiquitous in both males and females suggests that androgens are likely to have an impact in various tissues including the breast. Even though the actions of androgens are believed to oppose that of estrogens, the inhibitory effect of androgens on breast tissue depends largely on the relative tissue concentrations of both types of hormones as well as their receptor-mediated actions. This conclusion is consistent with two observations: (1) breasts do not undergo normal development in girls diagnosed with androgen-secreting adrenal tumors despite adequate circulating estrogens [68] and (2) development of gynecomastia in men treated with AR blockers for prostate cancer [69]. Clinically, a possible link has been established between AR expression and outcomes in certain subsets of breast cancer [70, 71]. Moreover, therapeutic application of androgens predated the selective estrogen receptor modulators in postmenopausal women with breast cancer [72, 73]. 

**Figure 5:**
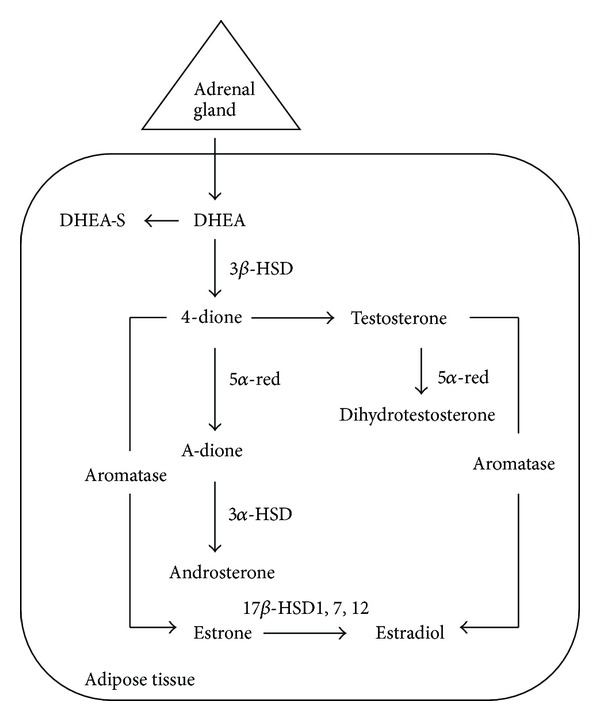
Sex steroidogenesis via the intracrine pathway. Androgens and estrogens synthesized in peripheral tissue including the breast require the presence of DHEA, an inactive precursor derived from the adrenal gland, and all of the appropriate enzymes. In addition to their synthesis, sex steroids are also inactivated in the same tissue thus minimizing widespread systemic effects. DHEA = dehydroepiandrosterone; DHEA-S = DHEA sulfated; 4-dione = androstenedione; A-dione = 5*α*-androstenedione; 3*α*-, 3*β*-, and 17*β*HSD = hydroxysteroid dehydrogenase; 5*α*-red = reductase.

Relative to ER*α*, the role of androgens in breast cancer growth and disease progression is less well understood. This level of uncertainty and, perhaps, appreciation persists in spite of the apparent correlation between tumor cell expression of AR in the majority of human breast cancers and various features of the disease mentioned earlier in this paper. The importance attached to this discordance necessitates a brief discussion of several salient issues. First, epidemiological studies have, on occasion, reached different conclusions with respect to androgens and breast cancer with the hormones either enhancing the protection against or increasing the risk for the disease [74]. One of the major reasons for this discrepancy relates to the validity of the correlation between disease risk and serum androgen concentrations [75]. Reliance on blood levels alone, however, obscures the potential importance of extragonadal synthesis of androgens (and estrogens). Identification of all the requisite enzymes including the 5*α*-reductases and both 3*β*- and 17*β*-hydroxysteroid dehydrogenases in peripheral tissue supports the relevance of a biological process known as intracrinology (Figure 5). That locally produced androgens can exert their actions in an intracrine manner may have added importance especially in postmenopausal women, who not only have a higher risk of breast cancer but who also rely on peripheral synthesis of nearly all sex hormones from adrenal-derived dehydroepiandrosterone (DHEA) [76]. Moreover, the best indicator of the total androgen reservoir as well as androgenic activity has been shown to be the conjugated metabolites of dihydrotestosterone (DHT), not the active hormone [76]. 

Second, results of *in vitro* studies are discordant even in AR+ breast cancer cell lines [77]. When exposed to DHT, a proliferative response occurred in both MDA-MB-453 cells (AR+/ER−) and MCF-7 (AR+/ER+) cell lines; however, an anti-proliferative effect was observed in two other AR+/ER+ cell lines, T47-D and ZR-75.l. One of the more intriguing explanations for the differential effect on proliferation in ER− and ER+ tumor cell lines may involve an interaction between AR and HER2 as both receptors are coexpressed in approximately 50% of ER− breast cancers [78]. In breast cancers classified as molecular apocrine subtype, androgen-induced proliferation involves activation of the extracellular signal-regulated-kinase-(ERK-) signal transduction pathway. However, the AR link to ERK has been shown to be dependent on transcriptional upregulation of the *HER2* gene. Hence, a functional feedback loop has been postulated in this type of breast cancer which includes AR, HER2, ERK, and an ERK transcription factor that binds to a promoter region on the *AR* gene [79]. What is also notable about this study is that a number of possible confounding causes for the divergent cellular responses such as cell density at time of drug exposure and serum used in the culture medium were eliminated by the meticulous methodology used by the investigators. Another plausible explanation for the discrepant results is the involvement of AR-independent pathways. Because androgens such as DHT can be metabolized (not aromatized) *in vitro*, one metabolite has been shown to bind ER*α* resulting in the proliferation of the MCF-7 cells [80]. It should also be mentioned that even though AR levels varied markedly between the different cell lines, receptor numbers alone cannot account for the divergent effects on proliferation. This conclusion is supported by the contrasting results observed in the two cell lines with the highest level of AR, MDA-MB-453, and ZR-75.l. In addition, despite a 6-fold lower AR expression, MCF-7 cells had a similar proliferative response as MDA-MB-453 cells. 

Even more perplexing is one other finding related to androgen action in MCF-7 breast cancer cells. While proliferation of MCF-7 breast cancer cells has been shown to occur even in the absence of estrogens, the proliferative effect may not be mediated through the androgen receptor pathway. In the presence of supra-physiological concentrations of DHT, nuclear translocation of both AR and ER occurred. That the stimulatory effect of DHT could have been mediated via ER was supported by increased expression of PR and the inability of antiandrogens to inhibit tumor cell growth [81]. This *in vitro *approach demonstrated that at physiological levels of DHT the androgen does not compete with estradiol for ER*α* binding. In addition, an intriguing *in vivo *correlate exists, one where breast tumor cells with negligible expression of ER*α* are exposed to high levels of androgens. In this clinically possible setting, it is rational to suggest that the rather promiscuous affinity of androgen, or an androgen metabolite [82], for the estrogen receptor provides a plausible explanation for tumors being characterized as ER−/PR+.

Third, clinical data indicate that expression of AR occurs more frequently than ER in breast tumor tissue. Interestingly, 10%–35% of triple negative and up to 60% of ER−/PR−/HER2+ breast cancers have been reported to express AR [71, 83–85]. However, unlike ER*α*, AR expression alone may not be predictive of response as anticancer benefits have been demonstrated with both androgens and anti-androgens. These confounding findings strongly suggest that other factors, including signaling through the epidermal growth factor receptor (EGFR) and HER2 pathways or activation of a key intermediary downstream kinase such as mitogen-activated protein kinase (MAPK), may contribute to the complex repertoire of androgen-mediated effects [86, 87]. The contribution of other signaling pathways has been nicely demonstrated in an ER*α*−/PR− breast cancer cell line engineered to express AR. While upregulation of the AR-target gene *p21* ^*WAF1*^ promoted tumor cell proliferation through the MAPK pathway, hyperactivation of MAPK by concurrent AR and EGFR-signaling pathways caused tumor growth suppression [88, 89]. 

Fourth, despite the frequency of AR expression in primary breast cancers, the function and role of the receptor may not be the same as in prostate cancer. This conclusion is supported by several findings regarding androgen signaling in both endocrine-related cancers. For instance, *AR* gene amplification is frequently observed in prostate cancer but not in breast cancer, despite receptor overexpression [90]. Whereas high AR expression has been correlated with poorer outcomes in prostate cancer, some investigators have found a direct correlation between AR expression and survival in patients with breast cancer [13, 91, 92]. Nonetheless, high AR expression may provide an advantage for both cancers especially since serum androgen and adrenal-derived DHEA levels, although markedly reduced by androgen-ablative therapy or aging, can be partially blunted by the process of intracrinology.

## 5. Hormone Receptor Milieu in Breast Cancer

Very compelling evidence support the belief that, in contrast to estrogen-induced proliferation of ER*α*+ tumor cells, tumor suppressive effects are mediated through ER*β* regardless of the second receptor that is expressed alone or in the presence of ER*α* [93]. This distinction may be clinically relevant to what has been traditionally labeled hormone receptor positive, and possibly, even hormone receptor negative breast cancer. One therapeutic implication relates to endoxifen, often regarded as the most potent metabolite of tamoxifen [94]. Exposure of MCF-7 (control) and ER*β*1-transfected MCF-7 cells to estrogen and endoxifen resulted in some striking between-cell differences. Unlike the rapid proteosomal degradation of ER*α* in control cells, stable accumulation of ER*β*1 was observed in the transfected cells. Notably, the ER*α* protein levels were also protected from enzymatic catabolism in the transfected cells, a finding supported by immunoprecipitation assays which indicated that the antiestrogen promoted formation of receptor heterodimers. In addition, endoxifen inhibited the upregulation of estrogen-responsive genes such as *cyclin D1* and *PR* in both cell lines. Of note also, significantly lower concentrations of the anti-estrogen (i.e., 40 nM compared to 1000 nM) were required for the same degree of inhibition in the ER*β*1-transfected cells. This finding suggested that the presence of ER*β*1 appeared to intensify the antagonistic effects of endoxifen. Though speculative, it is conceivable that the expression status of ER*β*1, which was not assessed in all of the CYP2D6 studies, could partially explain the conflicting predictive role of CYP2D6 pharmacogenomics and breast cancer outcomes [95]. Lastly, 17 biological pathways were found to be unique to either the control or the MCF-7 ER*β*1 cell lines. Two ER*α*-mediated pathways, one involving the regulation of G1/S cell cycle progression and the other cell migration, could be affected by endoxifen only if the cells coexpressed ER*β*1. Even though the gene expression pattern observed in the transfected cells could be linked to either ER homodimer, the degradation of ER*α* suggests that changes in the gene profile, as well as the enhanced antiestrogen effect of endoxifen, may result from the inhibition of ER*α*/ER*β*1 heterodimer.

Interesting also is the apparent paradox related to the role of estrogens in tumor cell survival and tumor cell death with the latter being mediated by mechanisms other than hormone deprivation. As certain as we were about tumor cell survival, both clinical and laboratory data unexpectedly demonstrated that, under certain circumstances, introduction of low doses of estrogens appears to have a protective effect on breast cancer [96, 97]. This “anti-tumor” effect was observed among surgically castrated postmenopausal women randomized to hormone replacement therapy as well as in a tamoxifen-resistant MCF-7 cell line. Notably, long-term estrogen deprivation appears to modulate apoptotic pathways of both normal and cancer cells either by inducing expression of proteins involved in the extrinsic cell-death pathway such as FasR/FasL or affecting regulatory components of the mitochondrial (i.e., intrinsic) apoptotic pathway including induction of the proapoptotic proteins Bax, Bak, and Bim [98]. While this may be the most reasonable explanation, it is also possible that actions mediated by ER*β*1 or AR could have modulated cell survival. What may be more intriguing about AR as a breast cancer target relates to a novel finding in ER−/AR+/HER2+ tumor samples [84]. Particularly alluring was the gene expression profile, one aspect of which indicated that AR-mediated signaling upregulated *WNT7B* transcription and activated *β*-catenin. Through their collaborative effort, AR and *β*-catenin were directly linked to androgen-induced *HER3 *expression and, indirectly, to the pernicious tumor characteristics imparted by the HER2/HER3 heterodimer [99]. As such, suppression of HER2 signaling as well as formation of the heterodimer could be achieved by AR inhibitors. 

Equally engaging is the concept that aberrations of the encoding genes may not only influence hormonal actions, but also breast cancer risk. That hormone insensitivity observed with other nuclear receptors has been positively linked to alterations in their respective genes, deletions, rearrangements, and point mutations in the *ER*α** gene could, vis-a-vis, also be associated with susceptibility to developing the disease. Indeed, even before data from the Human Genome and the HapMap projects were published, genotyping technology was being utilized to determine whether sequence variations or single-nucleotide polymorphisms (SNPs) in the *ER* genes were associated with disease risk. Two of the better characterized variants in the *ER*α** gene are *Pvu*II, a two-allele (1 and 0) restriction fragment-length polymorphism (RFLP) and *Xbal*. Even so, conflicting published data abound. For example, results of several studies indicated a significant, but not complete, correlation between the *Pvu*II genotype and ER*α*-positive breast cancers [100, 101]. In contrast, another study found that the prevalence of ER*α*-positive tumors was highest among women homozygous or heterozygous for the 0 (i.e., *Pvu*00 and *Pvu*01) allele and lowest in those harboring the* ER*α** gene *Pvu*II alleles [102]. Despite the contradictory data, the relatively weak overall correlation between RFLP genotype and ER*α* expression found in both studies suggests that this aberration in genomic sequence may not be linked to disease risk or receptor phenotype. The latter conclusion is supported by numerous other studies which found no frequency difference of the *Pvu*II polymorphism between breast cancer cases and controls [102–104]. Similar contrary findings have been reported for the *Xbal *polymorphism [101, 103, 104]. Polymorphisms of the *ER*β*1* gene have also been detected, and though there may be an association between the identified SNPs and disease risk, the data are way too preliminary to be of substantive value [105–107].

## 6. Conclusion

Embedded in the widely accepted dogma that estrogens are carcinogenic in breast tissue is the pivotal role of the estrogen receptor, specifically ER*α*. Nonetheless, accumulating evidence suggests that ER*β*1 and possibly AR also appear to have influential actions in the mammary gland. If the clinical relevance of especially ER*β*1 and AR can be firmly established, several new intrinsic subtypes of endocrine-sensitive breast cancers may emerge and subsequently undergo gene expression profiling. Unique in terms of their hormone dependency, each subtype may require a different hormonal approach in order to improve disease outcome. And while reference to ER+ (i.e., ER*α*+/PR−) breast cancer will still be appropriate, designation of specific double (i.e., ER*α*/ER*β*-variant, ER*α*/AR, and ER*β*-variant/AR) or triple (i.e., ER*α*, ER*β*-variant, and AR) hormone receptor-positive tumors may have greater clinical significance. Moreover, truly hormone receptor-negative breast cancers are apt to be less frequently diagnosed, incrementally more likely not to benefit from any type of hormonal intervention, and, perhaps, even be of better prognostic value.

Although ER*α* is one of the oldest tumor targets, clinical evidence validates the conclusion that estrogen-deprivation strategies is the most effective way to treat hormone-dependent breast cancer. And despite the translational achievement between laboratory and clinic with approval of agents targeting ER*α*, the expected concordance between construct inhibition and tumor eradication has, in some respects, been overestimated. The apparent incongruence suggests that mere identification of the target(s), regardless of its (their) purported functional importance, exaggerates our knowledge of tumor cell signaling pathways which ultimately controls cancer cell growth and survival. Until all hormone receptor components are fully understood, the optimal clinical impact of antihormonal therapies will not be fully realized.

## Figures and Tables

**Table 1:** Receptor-mediated effects and features in breast cancer.

	ER*α*	ER*β*	ER*α*/ER*β* heterodimer	AR
Proliferation	Stimulates (in the presence of E2 and coactivators)	Suppresses but may depend on variant (only ER*β*1 binds E2 with high affinity)	Data support suppression but may depend on ER*β* variant and density of each receptor subtype	Stimulates or suppresses (may depend on concurrent signaling pathways activated)

Apoptosis	Suppresses (in the presence of E2 and coactivators)	Promotes (but may depend on variant)	Data support promotion but may depend on ER*β* variant and density of each receptor subtype	Stimulates or suppresses (may depend on concurrent signaling pathways or variant)

*PR * gene	Activates	Represses activation	Represses activation	—

Prognostic factor	Yes	Uncertain but possible	Uncertain but possible	Uncertain but possible

ERE binding	Yes	Only ER*β*1, *β*ins, and ER*β*2 bind ERE	Yes	Androgen response element (ARE)

Dimerization with ER*α* and ER*β*1	Yes	Yes for wild type and *β*ins; ER*β*2 binds ER*α* only	—	No

Receptor variants	Best characterized is hER-alpha36	At least 5 (ER*β*1 (wild type), delta5, 18 a.a insert (*β*ins), ER*β*cx (ER*β*2), and *β*5)	—	At least 7 splice variants have been identified (AR-V1, AR-V2, AR-V3, AR-V4, AR-V5, AR-V6, and AR-V7) but many more likely to exist

AF-2	Independent	Dependent	—	—

Interaction with other signaling pathways	Yes	Yes	Yes	Yes

## References

[1] Boyd S (1900). On oophorectomy in cancer of the breast. *British Medical Journal*.

[2] Jensen VE, Jacobson IH, Walf AA, Frye AC (1962). Basic guides to the mechanism of estrogen action. *Recent Progress in Hormone Research*.

[3] Cole MP, Jones CT, Todd ID (1971). A new anti-oestrogenic agent in late breast cancer, an early clinical appraisal of ICI46474. *British Journal of Cancer*.

[4] Fisher B, Redmond C, Fisher ER, Caplan R (1988). Relative worth of estrogen or progesterone receptor and pathologic characteristics of differentiation as indicators of prognosis in node negative breast cancer patients: findings from national surgical adjuvant breast and bowel project protocol B-06. *Journal of Clinical Oncology*.

[5] Sørlie T, Perou CM, Tibshirani R (2001). Gene expression patterns of breast carcinomas distinguish tumor subclasses with clinical implications. *Proceedings of the National Academy of Sciences of the United States of America*.

[6] Paik S, Tang G, Shak S (2006). Gene expression and benefit of chemotherapy in women with node-negative, estrogen receptor-positive breast cancer. *Journal of Clinical Oncology*.

[7] Kuiper GGJM, Enmark E, Pelto-Huikko M, Nilsson S, Gustafsson J-Å (1996). Cloning of a novel estrogen receptor expressed in rat prostate and ovary. *Proceedings of the National Academy of Sciences of the United States of America*.

[8] Marotti JD, Collins LC, Hu R, Tamimi RM (2010). Estrogen receptor-*β* expression in invasive breast cancer in relation to molecular phenotype: results from the nurses’ health study. *Modern Pathology*.

[9] Miller WR, Anderson TJ, Dixon JM, Saunders PTK (2006). Oestrogen receptor *β* and neoadjuvant therapy with tamoxifen: prediction of response and effects of treatment. *British Journal of Cancer*.

[10] Honma N, Horii R, Iwase T (2008). Clinical importance of estrogen receptor-*β* evaluation in breast cancer patients treated with adjuvant tamoxifen therapy. *Journal of Clinical Oncology*.

[11] Engelsman E, Korsten CB, Persijn JP, Cleton FJ (1974). Proceedings: oestrogen and androgen receptors in human breast cancer. *British Journal of Cancer*.

[12] Park S, Koo J, Park HS (2010). Expression of androgen receptors in primary breast cancer.. *Annals of Oncology*.

[13] Agoff SN, Swanson PE, Linden H, Hawes SE, Lawton TJ (2003). Androgen receptor expression in estrogen receptor-negative breast cancer: immunohistochemical, clinical, and prognostic associations. *American Journal of Clinical Pathology*.

[14] Moinfar F, Okcu M, Tsybrovskyy O (2003). Androgen receptors frequently are expressed in breast carcinomas: potential relevance to new therapeutic strategies. *Cancer*.

[15] Mangelsdorf DJ, Thummel C, Beato M (1998). The nuclear receptor superfamily: the second decade. *Cell*.

[16] Cowley SM, Parker MG (1999). A comparison of transcriptional activation by ER*α* and ER*β*. *Journal of Steroid Biochemistry and Molecular Biology*.

[17] Truss M, Beato M (1993). Steroid hormone receptors: interaction with deoxyribonucleic acid and transcription factors. *Endocrine Reviews*.

[18] Dahlman-Wright K, Wright A, Gustafsson J, Carlstedt-Duke J (1991). Interaction of the glucocorticoid receptor DNA-binding domain with DNA as a dimer is mediated by a short segment of five amino acids. *Journal of Biological Chemistry*.

[19] Glass CK (1994). Differential recognition of target genes by nuclear receptor monomers, dimers, and heterodimers. *Endocrine Reviews*.

[20] Schwabe JWR, Chapman L, Finch JT, Rhodes D (1993). The crystal structure of the estrogen receptor DNA-binding domain bound to DNA: how receptors discriminate between their response elements. *Cell*.

[21] Schoenmakers E, Verrijdt G, Peeters B, Verhoeven G, Rombauts W, Claessens F (2000). Differences in DNA binding characteristics of the androgen and glucocorticoid receptors can determine hormone-specific responses. *Journal of Biological Chemistry*.

[22] Langley E, Zhou Z-X, Wilson EM (1995). Evidence for an anti-parallel orientation of the ligand-activated human androgen receptor dimer. *Journal of Biological Chemistry*.

[23] Kemppainen JA, Langley E, Wong C-I, Bobseine K, Kelce WR, Wilson EM (1999). Distinguishing androgen receptor agonists and antagonists: distinct mechanisms of activation by medroxyprogesterone acetate and dihydrotestosterone. *Molecular Endocrinology*.

[24] Zhou Z-X, Lane MV, Kemppainen JA, French FS, Wilson EM (1995). Specificity of ligand-dependent androgen receptor stabilization: receptor domain interactions influence ligand dissociation and receptor stability. *Molecular Endocrinology*.

[25] Li J, Fu J, Toumazou C, Yoon H-G, Wong J (2006). A role of the amino-terminal (N) and carboxyl-terminal (C) interaction in binding of androgen receptor to chromatin. *Molecular Endocrinology*.

[26] Doesburg P, Kuil CW, Berrevoets CA (1997). Functional in vivo interaction between the amino-terminal, transactivation domain and the ligand binding domain of the androgen receptor. *Biochemistry*.

[27] Brzozowski AM, Pike ACW, Dauter Z (1997). Molecular basis of agonism and antagonism in the oestrogen receptor. *Nature*.

[28] Sack JS, Kish KF, Wang C (2001). Crystallographic structures of the ligand-binding domains of the androgen receptor and its T877A mutant complexed with the natural agonist dihydrotestosterone. *Proceedings of the National Academy of Sciences of the United States of America*.

[29] Heery DM, Kalkhoven E, Hoare S, Parker MG (1997). A signature motif in transcriptional co-activators mediates binding to nuclear receptors. *Nature*.

[30] Jenster G, Van der Korput HAGM, Trapman J, Brinkmann AO (1995). Identification of two transcription activation units in the N-terminal domain of the human androgen receptor. *Journal of Biological Chemistry*.

[31] Smith CL, O’Malley BW (2004). Coregulator function: a key to understanding tissue specificity of selective receptor modulators. *Endocrine Reviews*.

[32] Lopez-Garcia J, Periyasamy M, Thomas SR (2006). ZNF366 is an estrogen receptor corepressor that acts through CtBP and histone deacetylases. *Nucleic Acids Research*.

[33] Ishizuka T, Lazar MA (2003). The N-COR/histone deacetylase 3 complex is required for repression by thyroid hormone receptor. *Molecular and Cellular Biology*.

[34] Webb P, Nguyen P, Kushner PJ (2003). Differential SERM effects on corepressor binding dictate ER*α* activity *in vivo*. *Journal of Biological Chemistry*.

[35] Bocchinfuso WP, Korach KS (1997). Mammary gland development and tumorigenesis in estrogen receptor knockout mice. *Journal of Mammary Gland Biology and Neoplasia*.

[36] Hewitt SC, Bocchinfuso WP, Zhai J (2002). Lack of ductal development in the absence of functional estrogen receptor *α* delays mammary tumor formation induced by transgenic expression of ErbB2/neu. *Cancer Research*.

[37] Todaro GJ, Rose TM, Spooner CE, Shoyab M, Plowman GD (1990). Cellular and viral ligands that interact with the egf receptor. *Seminars in Cancer Biology*.

[38] Kleinberg DL (1997). Early mammary development: growth hormone and IGF-1. *Journal of Mammary Gland Biology and Neoplasia*.

[39] Lin Z, Reierstad S, Huang C-C, Bulun SE (2007). Novel estrogen receptor-*α* binding sites and estradiol target genes identified by chromatin immunoprecipitation cloning in breast cancer. *Cancer Research*.

[40] Klaus A, Birchmeier W (2008). Wnt signalling and its impact on development and cancer. *Nature Reviews Cancer*.

[41] Roger P, Sahla ME, Mäkelä S, Gustafsson JÅ, Baldet P, Rochefort H (2001). Decreased expression of estrogen receptor *β* protein in proliferative preinvasive mammary tumors. *Cancer Research*.

[42] Chang EC, Frasor J, Komm B, Katzenellenbogen BS (2006). Impact of estrogen receptor *β* on gene networks regulated by estrogen receptor *α* in breast cancer cells. *Endocrinology*.

[43] Lazennec G, Bresson D, Lucas A, Chauveau C, Vignon F (2001). ER*β* inhibits proliferation and invasion of breast cancer cells. *Endocrinology*.

[44] Paruthiyil S, Parmar H, Kerekatte V, Cunha GR, Firestone GL, Leitmant DC (2004). Estrogen receptor *β* inhibits human breast cancer cell proliferation and tumor formation by causing a G2 cell cycle arrest. *Cancer Research*.

[45] Skliris GP, Leygue E, Curtis-Snell L, Watson PH, Murphy LC (2006). Expression of oestrogen receptor-*β* in oestrogen receptor-*α* negative human breast tumours. *British Journal of Cancer*.

[46] O’Neill PA, Davies MPA, Shaaban AM (2004). Wild-type oestrogen receptor beta (ER*β*1) mRNA and protein expression in tamoxifen-treated post-menopausal breast cancers. *British Journal of Cancer*.

[47] Jensen EV, Cheng G, Palmieri C (2001). Estrogen receptors and proliferation markers in primary and recurrent breast cancer. *Proceedings of the National Academy of Sciences of the United States of America*.

[48] Cremoux PD, Tran-Perennou C, Elie C (2002). Quantitation of estradiol receptors alpha and beta and progesterone receptors in human breast tumors by real-time reverse transcription-polymerase chain reaction: correlation with protein assays. *Biochemical Pharmacology*.

[49] Bièche I, Parfait B, Laurendeau I, Girault I, Vidaud M, Lidereau R (2001). Quantification of estrogen receptor *α* and *β* expression in sporadic breast cancer. *Oncogene*.

[50] Saji S, Sakaguchi H, Andersson S, Warner M, Gustafsson J-Å (2001). Quantitative analysis of estrogen receptor proteins in rat mammary gland. *Endocrinology*.

[51] Saji S, Omoto Y, Shimizu C (2002). Expression of estrogen receptor (ER) *β*cx protein in ER*α*-positive breast cancer: specific correlation with progesterone receptor. *Cancer Research*.

[52] Vladusic EA, Hornby AE, Guerra-Vladusic FK, Lupu R (1998). Expression of estrogen receptor *β* messenger RNA variant in breast cancer. *Cancer Research*.

[53] Palmieri C, Cheng GJ, Saji S (2002). Estrogen receptor beta in breast cancer. *Endocrine-Related Cancer*.

[54] Iwao K, Miyoshi Y, Egawa C, Ikeda N, Tsukamoto F, Noguchi S (2000). Quantitative analysis of estrogen receptor-alpha and -beta messenger RNA expression in breast carcinoma by real-time polymerase chain reaction. *Cancer*.

[55] Enmark E, Pelto-Huikko M, Grandien K (1997). Human estrogen receptor beta-gene structure, chromosomal localization, and expression pattern. *The Journal of Clinical Endocrinology & Metabolism*.

[56] Helguero LA, Faulds MH, Gustafsson J-Å, Haldosén L-A (2005). Estrogen receptors alfa (ER_*α*_) and beta (ER_*β*_) differentially regulate proliferation and apoptosis of the normal murine mammary epithelial cell line HC11. *Oncogene*.

[57] Pettersson K, Delaunay F, Gustafsson J-A (2000). Estrogen receptor *β* acts as a dominant regulator of estrogen signaling. *Oncogene*.

[58] Saji S, Hirose M, Toi M (2005). Clinical significance of estrogen receptor *β* in breast cancer. *Cancer Chemotherapy and Pharmacology*.

[59] Cowley SM, Hoare S, Mosselman S, Parker MG (1997). Estrogen receptors *α* and *β* form heterodimers on DNA. *Journal of Biological Chemistry*.

[60] Pettersson K, Grandien K, Kuiper GGJM, Gustafsson J-Å (1997). Mouse estrogen receptor *β* forms estrogen response element-binding heterodimers with estrogen receptor *α*. *Molecular Endocrinology*.

[61] Murphy LC, Peng B, Lewis A (2005). Inducible upregulation of oestrogen receptor-*β*1 affects oestrogen and tamoxifen responsiveness in MCF7 human breast cancer cells. *Journal of Molecular Endocrinology*.

[62] Papoutsi Z, Zhao C, Putnik M, Gustafsson J-Å, Dahlman-Wright K (2009). Binding of estrogen receptor *α*/*β* heterodimers to chromatin in MCF-7 cells. *Journal of Molecular Endocrinology*.

[63] Davies MPA, O’Neill PA, Innes H (2004). Correlation of mRNA for oestrogen receptor beta splice variants ER*β* 1, ER*β*2/ER*β*cx and ER*β*5 with outcome in endocrine-treated breast cancer. *Journal of Molecular Endocrinology*.

[64] Palmieri C, Lam EW, Mansi J The expression of ERbetacx in human breast cancer and the relationship to endocrine therapy and survival. *Clinical Cancer Research2004*.

[65] Moore JT, McKee DD, Slentz-Kesler K (1998). Cloning and characterization of human estrogen receptor *β* isoforms. *Biochemical and Biophysical Research Communications*.

[66] Hewitt SC, Korach KS (2000). Progesterone action and responses in the *α*ERKO mouse. *Steroids*.

[67] Matthews J, Wihlén B, Tujague M, Wan J, Ström A, Gustafsson J-Å (2006). Estrogen receptor (ER) *β* modulates ER*α*-mediated transcriptional activation by altering the recruitment of c-Fos and c-Jun to estrogen-responsive promoters. *Molecular Endocrinology*.

[68] Forsbach G, Güitrón-Cantú A, Vázquez-Lara J, Mota-Morales M, Díaz-Mendoza ML (2000). Virilizing adrenal adenoma and primary amenorrhea in a girl with adrenal hyperplasia. *Archives of Gynecology and Obstetrics*.

[69] Dobs A, Darkes MJM (2005). Incidence and management of gynecomastia in men treated for prostate cancer. *Journal of Urology*.

[70] Buchanan G, Birrell SN, Peters AA (2005). Decreased androgen receptor levels and receptor function in breast cancer contribute to the failure of response to medroxyprogesterone acetate. *Cancer Research*.

[71] Gucalp A, Traina TA (2010). Triple-negative breast cancer: role of the androgen receptor. *Cancer Journal*.

[72] Adair EF, Herrmann BJ (1946). The use of testosterone propionate in the treatment of advanced carcinoma of the breast. *Annals of Surgery*.

[73] Kennedy BJ (1958). Fluoxymesterone therapy in advanced breast cancer. *The New England Journal of Medicine*.

[74] Eliassen AH, Missmer SA, Tworoger SS (2006). Endogenous steroid hormone concentrations and risk of breast cancer among premenopausal women. *Journal of the National Cancer Institute*.

[75] Key TJ, Appleby P, Barnes I, Reeves G (2002). Endogenous sex hormones and breast cancer in postmenopausal women: reanalysis of nine prospective studies. *Journal of the National Cancer Institute*.

[76] Labrie F, Bélanger A, Cusan L, Candas B (1997). Physiological changes in dehydroepiandrosterone are not reflected by serum levels of active androgens and estrogens but of their metabolites: intracrinology. *Journal of Clinical Endocrinology and Metabolism*.

[77] Birrell SN, Bentel JM, Hickey TE (1995). Androgens induce divergent proliferative responses in human breast cancer cell lines. *Journal of Steroid Biochemistry and Molecular Biology*.

[78] Naderi A, Hughes-Davies L (2008). A functionally significant cross-talk between androgen receptor and ErbB2 pathways in estrogen receptor negative breast cancer. *Neoplasia*.

[79] Chia KM, Liu J, Francis GD, Naderi A (2011). A feedback loop between androgen receptor and erk signaling in estrogen receptor-negative breast cancer. *Neoplasia*.

[80] Roy R, Dauvois S, Labrie F, Belanger A (1992). Estrogen-stimulated glucuronidation of dihydrotestosterone in MCF-7 human breast cancer cells. *Journal of Steroid Biochemistry and Molecular Biology*.

[81] Zava DT, McGuire WL (1978). Androgen action through estrogen receptor in a human breast cancer cell line. *Endocrinology*.

[82] Hackenberg R, Turgetto I, Filmer A, Schulz K-D (1993). Estrogen and androgen receptor mediated stimulation and inhibition of proliferation by androst-5-ene-3*β*,17*β*-diol in human mammary cancer cells. *Journal of Steroid Biochemistry and Molecular Biology*.

[83] Ni M, Chen Y, Lim E (2011). Targeting androgen receptor in estrogen receptor-negative breast cancer. *Cancer Cell*.

[84] Niemeier LA, Dabbs DJ, Beriwal S, Striebel JM, Bhargava R (2010). Androgen receptor in breast cancer: expression in estrogen receptor-positive tumors and in estrogen receptor-negative tumors with apocrine differentiation. *Modern Pathology*.

[85] Park S, Koo J, Park HS (2010). Expression of androgen receptors in primary breast cancer. *Annals of Oncology*.

[86] Pignon J-C, Koopmansch B, Nolens G, Delacroix L, Waltregny D, Winkler R (2009). Androgen receptor controls EGFR and ERBB2 gene expression at different levels in prostate cancer cell lines. *Cancer Research*.

[87] Mellinghoff IK, Vivanco I, Kwon A, Tran C, Wongvipat J, Sawyers CL (2004). HER2/neu kinase-dependent modulation of androgen receptor function through effects on DNA binding and stability. *Cancer Cell*.

[88] Lu S, Jenster G, Epner DE (2000). Androgen induction of cyclin-dependent kinase inhibitor p21 gene: role of androgen receptor and transcription factor Sp1 complex. *Molecular Endocrinology*.

[89] Garay JP, Karakas B, Abukhdeir AM (2012). The growth response to androgen receptor signaling in ER*α*-negative human breast cells is dependent on p21 and mediated by MAPK activation. *Breast Cancer Research*.

[90] Visakorpi T, Hyytinen E, Koivisto P (1995). *In vivo* amplification of the androgen receptor gene and progression of human prostate cancer. *Nature Genetics*.

[91] Ogawa Y, Hai E, Matsumoto K (2008). Androgen receptor expression in breast cancer: relationship with clinicopathological factors and biomarkers. *International Journal of Clinical Oncology*.

[92] Rakha EA, El-Sayed ME, Green AR, Lee AHS, Robertson JF, Ellis IO (2007). Prognostic markers in triple-negative breast cancer. *Cancer*.

[93] Covaleda AMS, Van den Berg H, Vervoort J (2008). Influence of cellular ER*α*/ER*β* ratio on the ER*α*-agonist induced proliferation of human T47D breast cancer cells. *Toxicological Sciences*.

[94] Wu X, Subramaniam M, Grygo SB (2011). Estrogen receptor-beta sensitizes breast cancer cells to the anti-estrogenic actions of endoxifen. *Breast Cancer Research*.

[95] Regan MM, Leyland-Jones B, Bouzyk M (2012). CYP2D6 Genotype and tamoxifen response in postmenopausal women with endocrine-responsive breast cancer: the breast international group 1-98 trial. *Journal of the National Cancer Institute*.

[96] LaCroix AZ, Chlebowski RT, Manson JE (2011). Health outcomes after stopping conjugated equine estrogens among postmenopausal women with prior hysterectomy: a randomized controlled trial. *Journal of the American Medical Association*.

[97] Lewis JS, Meeke K, Osipo C (2005). Intrinsic mechanism of estradiol-induced apoptosis in breast cancer cells resistant to estrogen deprivation. *Journal of the National Cancer Institute*.

[98] Baselga J, Swain SM (2009). Novel anticancer targets: revisiting ERBB2 and discovering ERBB3. *Nature Reviews Cancer*.

[99] Lee-Hoeflich ST, Crocker L, Yao E (2008). A central role for HER3 in HER2-amplified breast cancer: implications for targeted therapy. *Cancer Research*.

[100] Hill SM, Fuqua SAW, Chamness GC, Greene GL, McGuire WL (1989). Estrogen receptor expression in human breast cancer associated with an estrogen receptor gene restriction fragment length polymorphism. *Cancer Research*.

[101] Cai Q, Shu X-O, Jin F (2003). Genetic polymorphisms in the estrogen receptor *α* gene and risk of breast cancer: results from the Shanghai breast cancer study. *Cancer Epidemiology Biomarkers and Prevention*.

[102] Yaich L, Dupont WD, Cavener DR, Parl FF (1992). Analysis of the *PvuII* restriction fragment-length polymorphism and exon structure of the estrogen receptor gene in breast cancer and peripheral blood. *Cancer Research*.

[103] Andersen TI, Heimdal KR, Skrede M, Tveit K, Berg K, Borresen A-L (1994). Oestrogen receptor (ESR) polymorphisms and breast cancer susceptibility. *Human Genetics*.

[104] Shin A, Kang D, Nishio H (2003). Estrogen receptor alpha gene polymorphisms and breast cancer risk. *Breast Cancer Research and Treatment*.

[105] Försti A, Zhao C, Israelsson E, Dahlman-Wright K, Gustafsson J-Å, Hemminki K (2003). Polymorphisms in the estrogen receptor beta gene and risk of breast cancer: no association. *Breast Cancer Research and Treatment*.

[106] Zheng SL, Zheng W, Chang B-L (2003). Joint effect of estrogen receptor *β* sequence variants and endogenous estrogen exposure on breast cancer risk in Chinese women. *Cancer Research*.

[107] Maguire P, Margolin S, Skoglund J (2005). Estrogen receptor beta (ESR2) polymorphisms in familial and sporadic breast cancer. *Breast Cancer Research and Treatment*.

